# Detecting *w*Mel *Wolbachia* in field-collected *Aedes aegypti* mosquitoes using loop-mediated isothermal amplification (LAMP)

**DOI:** 10.1186/s13071-019-3666-6

**Published:** 2019-08-15

**Authors:** Daniela da Silva Gonçalves, David J. Hooker, Yi Dong, Nathan Baran, Peter Kyrylos, Iñaki Iturbe-Ormaetxe, Cameron P. Simmons, Scott L. O’Neill

**Affiliations:** 1 World Mosquito Program, Institute of Vector-Borne Disease, Monash University, 12 Innovation Walk, Clayton, VIC 3800 Australia; 2 Oxford University Clinical Research Unit, Hospital for Tropical Diseases, Ho Chi Minh City, Vietnam

**Keywords:** *w*Mel, *Wolbachia*, LAMP, Diagnostics

## Abstract

**Background:**

The World Mosquito Program uses *Wolbachia pipientis* for the biocontrol of arboviruses transmitted by *Aedes aegypti* mosquitoes. Diagnostic testing for *Wolbachia* in laboratory colonies and in field-caught mosquito populations has typically employed PCR. New, simpler methods to diagnose *Wolbachia* infection in mosquitoes are required for large-scale operational use.

**Methods:**

Field-collected *Ae. aegypti* mosquitoes from North Queensland were tested using primers designed to detect the *Wolbachia wsp* gene, specific to the strain *w*Mel. The results were analysed by colour change in the reaction mix. Furthermore, to confirm the efficiency of the LAMP assay, the results were compared to the gold-standard qPCR test.

**Results:**

A novel loop-mediated isothermal amplification (LAMP) colorimetric test for the *w*Mel strain of *Wolbachia* was designed, developed and validated for use in a high-throughput setting. Against the standard qPCR test, the analytical sensitivity, specificity and diagnostic metrics were: sensitivity (99.6%), specificity (92.2%), positive predictive value (97.08%) and negative predictive value (99.30%).

**Conclusions:**

We describe an alternative, novel and high-throughput method for diagnosing *w*Mel *Wolbachia* infections in mosquitoes. This assay should support *Wolbachia* surveillance in both laboratory and field populations of *Ae. aegypti*.

**Electronic supplementary material:**

The online version of this article (10.1186/s13071-019-3666-6) contains supplementary material, which is available to authorized users.

## Background

Arboviral diseases transmitted by the mosquito *Aedes aegypti* such as dengue, chikungunya and Zika constitute a significant burden to human health and economic development worldwide [1, 2]. This is reflected in the nomination by the World Health Organisation of dengue as one of the top ten global health threats in 2019. There is an urgent need for novel and efficient strategies to control these diseases [3].

The World Mosquito Program (WMP, https://www.worldmosquitoprogram.org, formerly known as the Eliminate Dengue Program) has developed a novel arboviral disease biocontrol strategy utilising the endosymbiotic bacterium *Wolbachia pipientis*. This maternally transmitted bacterium [4] is found in 40–60% of insect species worldwide [5–7]. WMP reported the successful introduction of the *w*Mel strain of *Wolbachia* into *Ae. aegypti* in northern Australia since 2011 [8]. Subsequently, numerous studies demonstrated that the presence of *w*Mel reduces dengue virus [9], Zika [10], chikungunya virus [11] yellow fever [11] and Mayaro virus [12] infection and replication and, in turn the virus transmission potential of the mosquito [13, 14]. *w*Mel has been established in field populations of *Ae. aegypti* in five countries and it has been expanded to more than ten worldwide [15]. The mitigation of local dengue outbreaks in northern Australia [16] following the establishment of *w*Mel is consistent with the laboratory and modelling expectations of this intervention [17].

A duplex TaqMan^TM^ qPCR assay has been considered the gold-standard reference method for diagnosing *Wolbachia* infection in mosquitoes [5, 18]. The advantages of qPCR are clear: it is able to detect multiple genes of interest, produce quantitative or qualitative data [19] and is scalable. However, these advantages are significantly offset by high initial start-up costs and on-going maintenance of equipment, and the complexity of interpreting threshold values and amplification curves requires extensive training [20]. This is exacerbated in low-resource settings where the sourcing of laboratory equipment can be challenging, and technical expertise is not readily available. Given the rapid expansion of the World Mosquito Program [21, 22], there is a need to develop diagnostic tools suitable for resource limited settings.

Loop-mediated isothermal amplification (LAMP) [23, 24] is a technology potentially suitable for *Wolbachia* diagnostics in resource-limited laboratories [25, 26]. LAMP has been adapted as a nucleic acid test with multiple direct and indirect methods to detect several pathogens [27–30]. Hence the purpose of this study was to develop and validate the diagnostic accuracy of a colorimetric LAMP assay for the *w*Mel strain of *Wolbachia*.

## Methods

### Mosquito samples

Field-collected *Ae. aegypti* mosquitoes (*n* = 3585) were collected from BG-Sentinel traps during and after *Wolbachia* establishment in Cairns, Townsville and Innisfail, Australia, over an eight-month period from June 2017. The DNA from each mosquito was individually crudely extracted in the laboratory as previously described [31] before being tested by both TaqMan^TM^ qPCR [32] and LAMP assays.

### *w*Mel LAMP reactions

LAMP primers (Integrated DNA Technologies, Singapore, Singapore) were designed to detect the *wsp* gene from *w*Mel and *w*MelPop-CLA strains using the software LAMP Designer 1.02 (PREMIER Biosoft International, Horsham, UK). Individual reaction consisted of 2X WarmStart^®^ Colorimetric LAMP Master Mix (New England BioLabs, Ipswich, USA; Cat# M1800S), primers according to the manufacturer recommendation (Table 1), and 1 μl of target DNA in a total reaction volume of 17 μl. Reactions for individual samples were performed in 96-well PCR plates (LabAdvantage, Tingalpa, Australia; 96-well PCR plates, full skirt, clear). Plates were incubated in a thermocycler (BioRad C1000) at 65 °C for 30 min then held at 12 °C until scoring. Within one hour after incubation, colour changes of individual samples were recorded where pink indicates negative, yellow as positive and orange as equivocal (see Additional file 1: Figure S1). Results were interpreted by the naked eye directly from the reaction plates and also captured with a smartphone for data storage.

**Table 1:** LAMP primers targeting *w*Mel and *w*MelPop *Wolbachia* strains

Primer name	Primer sequence (5′–3′)	Length (bp)	Final primer concentrations (µM)
FIP_wMel/wPop	TGTATGCGCCTGCATCAGCTTCGGTTCTTATGGTGCTAA	39	1.6
BIP_wMel/wPop	GCAGAAGCTGGAGTAGCGTTGTGTCATGCCACTTAGATGG	40	1.6
F3_wMel/wPop	TGATGTAACTCCAGAAGTCA	20	0.2
B3_wMel/wPop	CTTATTGGACCAACAGGATCG	21	0.2
LpF_wMel/wPop	AGCCTGTCCGGTTGAATT	18	0.4
LpB_wMel/wPop	CAGTCTTGTTATCCCAGTGAGT	22	0.4

### *w*Mel LAMP assay performance

#### Target specificity

To assess the specificity of the LAMP assay for *w*Mel, multiple *Wolbachia* trans-infected *Ae. aegypti* lines (*w*Mel, *w*MelPop-CLA, *w*Ri, *w*Pip and *w*AlbB) were tested, in addition to two tetracycline-treated lines (*w*Mel.tet and *w*AlbB.tet). For each mosquito line, three mosquitos were tested and three technical replicates were performed, totalling nine reactions per line. Mosquitoes were reared and maintained as described [32], DNA was crudely extracted as previously described [31] and tested in the *w*Mel LAMP assay.

#### Diagnostic performance comparison

Field-collected samples were tested using both the *w*Mel LAMP and the reference TaqMan^TM^ qPCR assays [32]. The qPCR assay was modified by the use of an LC-640-tagged *wsp* gene probe (fluorescence bandwidth 618–660 nm) to provide improved separation from the FAM-tagged housekeeping gene *Rps17* (fluorescence bandwidth 465–510 nm). Both the qPCR and the LAMP results were analysed in blinded fashion, i.e. the interpretation of each assay was performed blind to the outcome of the other assay. The LAMP assay diagnostic specificity, sensitivity, positive predictive value (PPV) and negative predictive value (NPV) calculations were performed with any equivocal samples (orange colour) being excluded from the calculations. All statistical analysis and calculations were performed using Medcalc (https://www.medcalc.org/calc/diagnostic_test.php).

## Results

### *w*Mel LAMP assay performance

#### Target specificity

The *w*Mel LAMP assay consistently detected only the *wsp* target sequence from the *w*Mel strain in *Ae. aegypti*, and did not amplify the other six *Wolbachia* strains tested (Additional file 2: Figure S2).

#### Diagnostic performance on field-caught mosquitoes

A total of 3585 individual field-collected adult mosquitoes, sampled between June 2017 to February 2018 from Cairns, Townsville and Innisfail, were tested by both TaqMan^TM^ qPCR and LAMP and the comparison between the results from each assay is shown in Table 2. Amongst the field-caught mosquitoes, 24 (0.7%) were found to be non-*Ae. aegypti* due to non-amplification of *Ae. aegypti* housekeeping *Rps17* gene in duplex TaqMan^TM^ qPCR assay, and hence excluded from the analysis.

**Table 2:** *w*Mel LAMP positivity and negativity compared to qPCR

	qPCR-positive	qPCR-negative	Total
LAMP-positive	2327	70	2397
LAMP-negative	8	1128	1136
LAMP-equivocal	2	26	28
Total	2337	1224	3561

Relative to the qPCR reference method, LAMP false positives were more likely (*n* = 70) to occur than false negatives (*n* = 8). Equivocal LAMP results were more likely to result from qPCR-negative samples than qPCR-positive samples. LAMP increased the estimation of *w*Mel positivity by 2%, with an additional 1% of total samples producing equivocal results. The sensitivity of LAMP assay was close to 100% (Table 3) and this parameter was confirmed when performed in serially diluted field samples up to 1:1000 (data not shown). Also, *w*Mel LAMP diagnostic had high positive and negative predictive values in relation to the *w*Mel TaqMan^TM^ qPCR (Table 3).

**Table 3:** LAMP diagnostic parameters of LAMP-qPCR parallel testing

Diagnostic parameter	Value	95% CI
Specificity	94.16	92.67–95.42
Sensitivity	99.66	99.33–99.85
Accuracy	97.79	97.25–98.25
Positive predictive value	97.08	96.36–97.66
Negative predictive value	99.30	98.60–99.65

*Abbreviation*: CI, confidence interval

## Discussion

TaqMan^TM^ qPCR has been a mainstay for diagnosing *Wolbachia* infection in mosquitoes despite utilising expensive reagents and sophisticated equipment that require specialised training and maintenance [20]. Previous work has shown that there is potential to use LAMP for detecting *Wolbachia* in *Ae. aegypti,* either for any strain, targeting the *16S* rRNA gene [25], or specifically targeting the *wsp* gene of the strains *w*AlbB and *w*Pip [26]. Here, we have taken this framework and built on it by utilising a pH indicator that possesses the same characteristics but gives a greater resolution to differentiate between positive and negative results. The colorimetric LAMP assay in this study is an attractive candidate to replace qPCR because it does not require sophisticated equipment, is qualitative in nature, can easily be analysed by visual inspection and can be more cost-effective [33–35]. In addition, LAMP has been shown to be a reliable and robust assay across a range of DNA matrices [36] making it ideal for field-caught mosquito homogenates that can be highly variable. A small number of results were scored as false negatives. These could be explained by pipetting errors, or the presence of inhibitors of DNA amplification. Inhibitors such as EDTA, or human blood in blood-fed female mosquitoes, could block enzyme activity [37]. The frequency of false negatives was very low (0.22%), and does not affect the robustness of our assay.

When considering the implementation of colorimetric LAMP as the primary diagnostic method for monitoring the establishment of *w*Mel, certain trade-offs should be recognised. First, compared to qPCR there may be an increased likelihood of contamination due to the high amplification efficiency of LAMP [23, 38]. Secondly, as the colorimetric LAMP assay is a single target nucleic acid test, it relies on entomologists to accurately identify *Ae. aegypti* mosquitoes from other species and insects that might be collected from the field. Thirdly, despite its robustness, the LAMP assay can produce equivocal results occasionally, presenting as wells with varying hues of the colour orange. However, equivocal results were rare (typically 1% of the samples) and did not significantly impact on the predictive ability of the assay. In general, this rate of equivocal findings should not adversely affect the chronological and geographical picture of *Wolbachia* establishment. Finally, the *w*Mel LAMP assay is scored visually which may be subject to interpretation bias. To avoid possible visual biases, a smartphone application has been developed to conveniently and reliably score positivity and negativity, and this can promote consistency across multiple and international settings.

## Conclusions

In conclusion, the *w*Mel LAMP assay described here was sensitive, specific and suitable for high throughput application. With these results, we believe the assay is an appropriate tool to monitor the progress of *w*Mel *Wolbachia* establishment in field *Ae. aegypti* populations worldwide in order to protect local communities from mosquito-borne diseases.

## Additional files

**Additional file 1: Figure S1.** Example of colorimetric LAMP result interpretation. Results are scored based on colour change. Samples (1) and (2) in yellow are positive for *w*Mel *Wolbachia*; (3) and (4) in pink are negative; and (5) and (6) in orange are considered equivocal.

**Additional file 2: Figure S1.** Specificity of the *w*Mel LAMP assay. LAMP reactions were performed using a number of *Ae. aegypti* lines transinfected with different *Wolbachia* strains per column, as follows: (1) *w*Mel-infected, field-collected; (2) *w*Mel, purified gDNA; (3) *w*AlbB; (4) *Ae. aegypti* tetracycline treated (without *w*AlbB); (5) *w*MelPop-CLA; (6) *w*Pip; (7) *w*Ri; (8) *w*MelCS; (9) *Ae. aegypti* tetracycline treated (without *w*Mel); (10) wild type uninfected *Ae. aegypti* from Townsville, Australia; (11) water; and (12) extraction buffer negative control. Eight technical replicates were run for controls.

## Data Availability

All relevant data generated or analysed during this study are included in this published article and its additional files.
