# Supplementary figures and images for: Detecting wMel Wolbachia in field-collected Aedes aegypti mosquitoes using loop-mediated isothermal amplification (LAMP)
Source: Parasit Vectors. 2019 Aug 15;12:404. doi: 10.1186/s13071-019-3666-6 (PMC6694616; doi:10.1186/s13071-019-3666-6)

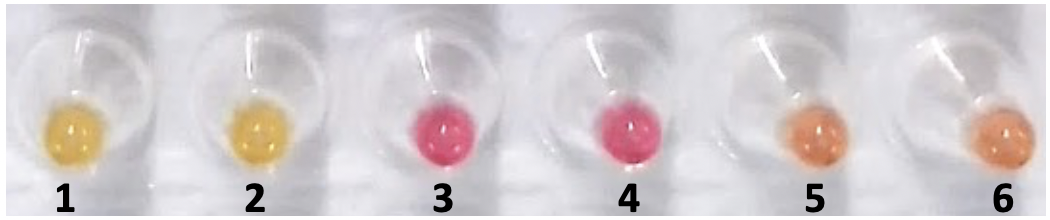

Supplement: Supplementary file 1 — Additional file 1: Figure S1. Example of colorimetric LAMP result interpretation. Results are scored based on colour change. Samples (1) and (2) in yellow are positive for wMel Wolbachia; (3) and (4) in pink are negative; and (5) and (6) in orange are considered equivocal. [file 13071_2019_3666_MOESM1_ESM.png]

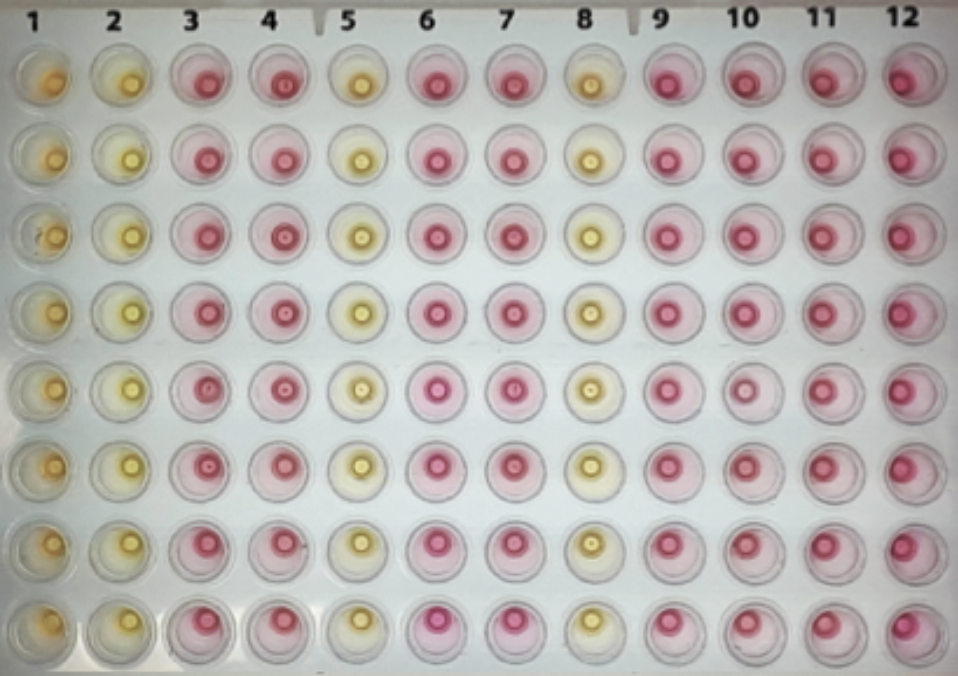

Supplement: Supplementary file 2 — Additional file 2: Figure S1. Specificity of the wMel LAMP assay. LAMP reactions were performed using a number of Ae. aegypti lines transinfected with different Wolbachia strains per column, as follows: (1) wMel-infected, field-collected; (2) wMel, purified gDNA; (3) wAlbB; (4) Ae. aegypti tetracycline treated (without wAlbB); (5) wMelPop-CLA; (6) wPip; (7) wRi; (8) wMelCS; (9) Ae. aegypti tetracycline treated (without wMel); (10) wild type uninfected Ae. aegypti from Townsville, Australia; (11) water; and (12) extraction buffer negative control. Eight technical replicates were run for controls. [file 13071_2019_3666_MOESM2_ESM.png]
